# Syphilis screening coverage and positivity by HIV treatment status among South African pregnant women enrolled in the 2019 antenatal HIV sentinel survey

**DOI:** 10.1038/s41598-023-32456-0

**Published:** 2023-04-01

**Authors:** Tendesayi Kufa, Selamawit Woldesenbet, Mireille Cheyip, Kassahun Ayalew, Ranmini Kularatne, Samuel Manda, Carl Lombard, Adrian Puren

**Affiliations:** 1 Centre for HIV and STIs, National Institute for Communicable Diseases, Johannesburg, South Africa; 2 School of Public Health, University of the Witwatersrand, 1 Modderfontein Road, Sandringham, Johannesburg, South Africa; 3 Centers for Disease Control and Prevention, Pretoria, South Africa; 4 Department of Clinical Microbiology and Infectious Diseases, School of Pathology, University of the Witwatersrand, Johannesburg, South Africa; 5 Biostatistics Research Unit, South African Medical Research Council, Pretoria, South Africa; 6 Department of Statistics, University of Pretoria, South Africa Medical Research Council, Pretoria, South Africa; 7 Biostatistics Research Unit, South African Medical Research Council, Cape Town, South Africa; 8 Department of Virology, School of Public Health, University of the Witwatersrand, Johannesburg, South Africa; 9 Division of Epidemiology and Biostatistics, Department of Global Health, University of Stellenbosch, Cape Town, South Africa

**Keywords:** Epidemiology, Population screening

## Abstract

We describe coverage of maternal syphilis screening, syphilis positivity, coverage of treatment and their association with maternal HIV infection and antiretroviral treatment (ART) status among pregnant women attending South African antenatal clinics. The 2019 antenatal care sentinel survey was a cross-sectional survey conducted from 1 October to 15 November 2019 at 1589 sentinel sites in all nine provinces of the country and aimed to enrol 36,000 pregnant women ages 15–49 years regardless of HIV, ART or syphilis status. Data collection procedures included obtaining written informed consent, a brief interview, medical record review and blood specimen collection. Completed data collection forms and specimens were sent to designated regional laboratories for data capture and HIV serology testing. Data analysis determined four outcomes i) syphilis screening coverage ii) syphilis positivity iii) coverage of any treatment and iv) with Benzathine penicillin G (BPG). Multivariable logistic regression models with or without interaction between HIV infection and ART status with province were used to determine factors associated with syphilis positivity. Of the 41 598 women enrolled, 35 900 were included in the analysis for syphilis screening coverage. The weighted syphilis screening coverage was 96.4% [95% Confidence Interval (CI) 95.9–96.7%] nationally and was lowest among HIV positive women not on ART at 93.5% (95% CI 92.2–94.5%). Syphilis positivity was 2.6% (95% CI 2.4–2.9%) nationally. Among those who were syphilis positive, 91.9% (95% CI 89.8–93.7%) had documentation of syphilis treatment status, of whom 92.0% (95% CI 89.8–93.9%) were treated, with the majority treated with one or more doses of BPG [92.2% (95% CI 89.8–94.3%)]. HIV-positive women, not on ART [adjusted odd ratio (aOR) 2.24 (95% 1.71–2.93)] and those on ART [aOR 2.25 (95% CI 1.91–2.64)] were more likely to be syphilis positive compared to those who were HIV negative. The national syphilis screening coverage met the global screening target of 95%. Syphilis positivity was higher among HIV positive women compared to negative women. Introduction of rapid syphilis testing and ensuring a universal supply of appropriate treatment for syphilis will reduce the likelihood of mother-to-child transmission of syphilis.

## Introduction

Left undetected or untreated, maternal syphilis can lead to transmission of syphilis infection to the unborn child. This phenomenon—termed mother to child transmission of syphilis (MTCTs)—can lead to adverse pregnancy outcomes such as still-birth, spontaneous abortion, preterm birth, low birthweight, neonatal deaths as well as symptomatic or asymptomatic congenital infection in the neonate^1,2^. Studies have reported that in 50- 80% of mothers with syphilis, adverse birth outcomes will result depending on the stage of infection and how early during pregnancy syphilis is detected and treated^3,4^. Maternal syphilis has also been shown to facilitate transmission of HIV from mother to child, increasing risk of transmission of HIV 2–2.5 times^5,6^. Maternal HIV- syphilis co-infection has been associated with poorer birth outcomes compared to syphilis or HIV infections alone^7^.

In 2016, it was estimated that there were 988 000 cases of active maternal syphilis globally with close to 60% occurring in sub-Saharan Africa. These maternal syphilis infections were associated with 661 000 cases of mother-to-child transmission of syphilis including 143 000 fetal deaths, 61 000 neonatal deaths, 41 000 preterm or low birthweight births and 109 000 clinical congenital infections^8^. The mother-to-child transmission of syphilis is preventable through the screening of pregnant women and treatment of those found to have the infection. The use of treponemal and non-treponemal serological tests within laboratories and as point of care tests allows the detection of women with past or current syphilis^1^. Screening is recommended at first antenatal care (ANC) booking and again around 32–34 weeks of gestation in order to detect infections acquired during pregnancy^9^. Three once weekly doses of Benzathine penicillin G (BPG) is the standard of care for the treatment of maternal syphilis with the first dose given at least 30 days prior to delivery considered sufficient to prevent MTCTs^9^.

The global strategy for the triple elimination of MTCT of HIV, syphilis and hepatitis requires that countries ensure that at least 95% of pregnant women attend antenatal care, 95% and 90% are screened for HIV/ syphilis and hepatitis B with 95% and 90% of those who are positive for HIV/syphilis and hepatitis B respectively are treated in order to prevent MTCT^1^. Prior to 2017, syphilis prevalence was measured annually during ANC HIV surveys until 2011 and thereafter every 2–4 years while coverage of screening and treatment were not measured previously. During the 2017 national antenatal care HIV prevalence survey (ANC survey), coverage of the maternal syphilis screening during pregnancy in South Africa was estimated at 96.1%. However, data on syphilis prevalence and coverage of treatment were not collected in this survey^10^. From the earlier surveys, syphilis prevalence among pregnant women was known to vary by province and HIV status and from the HIV surveys, coverage of HIV treatment also varied greatly by province. HIV prevalence was highest in the KwaZulu Natal, Mpumalanga, Eastern Cape and Gauteng (30.2–44.4%)—while syphilis prevalence was highest in Free State, KwaZulu Natal, North West and Northern Cape (2.2–4.6%), provinces which generally have lower HIV prevalence^11^. Knowledge of distribution of syphilis prevalence by province and HIV status will be required for planning of interventions around HIV and syphilis prevention and testing such as dual HIV/ syphilis testing.

Since 2014, South Africa and other countries reported shortages of BPG^12^. As a result of these shortages, there have been concerns that individuals with syphilis would go untreated, resulting in increased transmission of syphilis in the general population and to pregnant women and their unborn children. The 2019 ANC survey collected data on syphilis screening, prevalence of positive results and treatment among the syphilis positive based on record review and abstraction of data. We present data on coverage of maternal syphilis screening, syphilis positivity and coverage of treatment and their association with maternal HIV infection and ART treatment status.

## Results

### Study flow and coverage of syphilis screening

**Figure 1:**
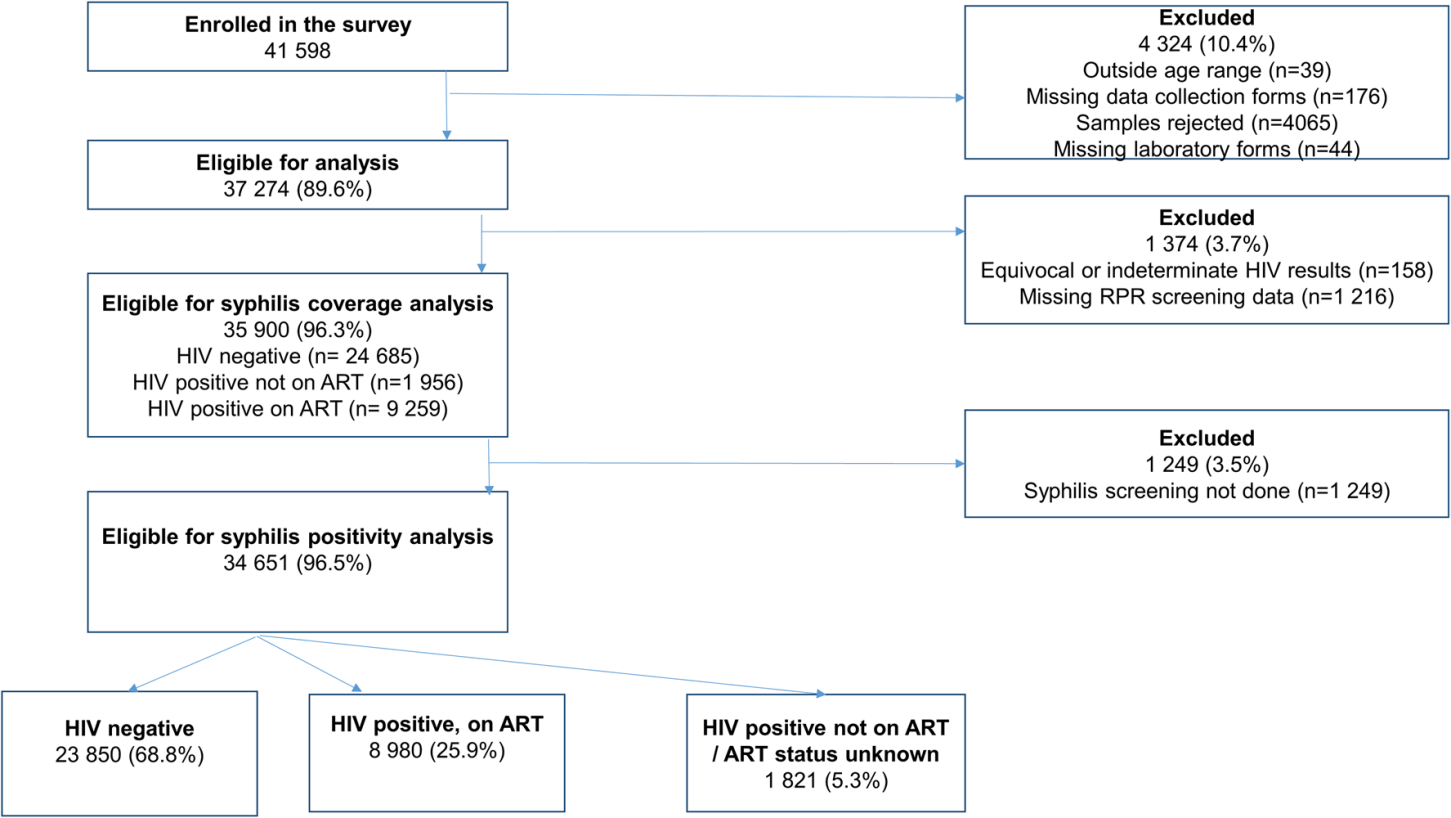
Study flow.

A total of 41 598 pregnant women were interviewed during the survey period. Of these, 4 324 (10.4%) were initially excluded because they fell out of the age range (15–49 years) (n = 39), had missing data collection forms (n = 176), specimens for HIV testing rejected in the laboratory, largely because of hemolysis (n = 4 065), and missing laboratory request forms (n = 44). Of the remaining 37 274, 158 (0.4%) were further excluded because they had missing HIV test results and 1216 (3.3%) were excluded because they had missing syphilis screening data. This resulted in 35 900 (96.3%) participants included in the analysis of coverage of syphilis screening (Fig. 1).

### Description of women included in the coverage analysis

Of the 35 900 women eligible for inclusion in the analysis for syphilis coverage, 24 685 (68.8%) were HIV negative, 1 956 (5.4%) were HIV positive but not taking ART or ART history was not documented and 9 259 (25.8%) were HIV positive and taking ART. Table 1 describes the demographic and clinical characteristics of the pregnant women included in the syphilis coverage analysis. The median age of the women was 26 years [interquartile range (IQR) 22–31 years] with 13 548 (40.1%) aged 15–24 years. The majority of women were of black African ethnicity, attended school up to secondary level, not living with the partner who fathered their child but were in a relationship with them and were enrolled from sentinel sites in the urban areas of Gauteng and KwaZulu-Natal. Just over two thirds (67.9%) were attending a follow up visit as opposed to a booking visit and 55.1% were in the second trimester of pregnancy with a median parity of 1 (interquartile range [IQR] 0–2) and gravity of 2 (IQR 1–3).

**Table 1:** Characteristics of eligible*pregnant women enrolled in the 2019 ANC HIV survey overall and by HIV/ART status, N = 35,900.

Variable	HIV negative 24 685 (68.8%)	HIV positive on ART 9 259 (25.8%)	HIV positive, not on ART 1 956 (5.5%)	Overall (unweighted) 35 900 (100%)	Overall weighted %
*Age category (years), n (%)*					
15–19	3872 (15.7)	317 (3.4)	144 (7.4)	4333 (12.1)	11.4
20- 24	7333 (29.7)	1449 (15.7	433 (22.1)	9215 (25.7)	25.4
25- 29	5813 (23.6)	2529 (27.3)	543 (27.8)	8885 (24.8)	24.8
30- 34	3704 (15.0)	2464 (26.6)	394 (20.1	6562 (18.3)	18.4
35- 49	2149 (8.7)	1872 (20.2)	241 (12.3)	4262 (11.9	11.8
Missing	1814 (7.4)	628 (6.8)	201 (10.3)	2643 (7.4)	8.3
*Population group*					
Black	20,822 (84.4)	8881 (95.9)	1835 (93.6)	31,538 (87.8)	88.8
Asian	109 (0.4)	18 (0.2)	6 (0.3)	133 (0.4)	0.4
Mixed race	3311 (13.4)	294 (3.2)	94 (4.8)	3699 (10.3)	9.3
White	200 (0.8)	11 (0.1)	6 (0.3)	217 (0.6)	0.7
Other	153 (0.6)	20 (0.2)	5 (0.3)	178 (0.5)	0.5
Missing	90 (0.4)	35 (0.4)	10 (0.5)	135 (0.4)	0.4
*Education*					
None	210 (0.9)	115 (1.2)	19 (1.0)	344 (1.0)	1.0
Primary	2543 (10.3)	1144 (12.4)	247 (12.6)	3934 (11.0)	11.4
Secondary	18,163 (73.6)	7077 (76.4)	1440 (73.6)	26,680 (74.3)	73.1
Tertiary	3594 (15.6)	857 (9.3)	228 (11.7)	4679 (13.0)	13.7
Missing	175 (0.7)	66 (0.7)	22 (1.1)	263 (0.7)	0.8
*Relationship with partner who fathered current pregnancy*					
Married	4556 (18.5)	1297 (14.0)	287 (14.7)	6140 (17.1)	17.6
Living together as married	6142 (24.9)	2716 (29.4)	524 (26.8)	9392 (26.2)	27.7
Not living together but in a relationship	12,915 (52.3)	4802 (51.9)	1038 (53.1)	18,755 (52.2)	50.2
No relationship	776 (3.1)	319 (3.5)	65 (3.3)	1160 (3.2)	3.2
Missing	296 (1.2)	115 (1.2)	42 (2.2)	453 (1.3)	1.3
*Age difference with partner who fathered current pregnancy*					
< 5 years	12,971 (52.6)	4645 (50.2)	929 (47.5)	18,545 (51.7)	51.1
≥ 5 years	9501 (38.5)	3720 (40.2)	820 (41.9)	14,041 (39.1)	39.7
Unknown	1503 (6.1)	641 (6.9)	140 (7.2)	2284 (6.4)	6.4
Missing	710 (2.9)	253 (2.7)	67 (3.4)	1030 (2.9)	2.9
*Province*					
Eastern Cape	3453 (14.0)	1582 (17.1)	411 (21.0)	5446 (15.2)	9.9
Free State	1883 (7.6)	779 (8.4)	142 (7.3)	2804 (7.8)	4.9
Gauteng	3701 (15.0)	1125 (12.2)	353 (18.1)	5179 (14.4)	25.6
KwaZulu-Natal	4860 (19.7)	2972 (32.1)	408 (20.9)	8240 (23.0)	21.1
Limpopo	2355 (9.5)	454 (4.9)	127 (6.5)	2936 (8.2)	10.5
Mpumalanga	1939 (7.9)	894 (9.7)	177 (9.1)	3010 (8.4)	7.4
Northern Cape	1319 (5.3)	260 (2.8)	74 (3.8)	1653 (4.6)	2.1
North West	2043 (8.3)	645 (7.0)	118 (6.0)	2806 (7.8)	7.1
Western Cape	3132 (12.7)	548 (5.9)	146 (7.5)	3826 (10.7)	11.4
*Geo-location*					
Rural	7853 (31.8)	3114 (33.6)	566 (28.9)	11,533 (32.1)	29.3
Peri-urban	2008 (8.1)	808 (8.7)	182 (9.3)	2998 (8.4)	7.8
Urban	14,824 (60.1)	5337 (57.6)	1208 (61.8)	21,369 (59.5)	62.9
*ANC visit*					
Follow up	15,837 (64.2)	6877 (74.3)	670 (34.3)	23,384 (65.1)	64.1
First ANC visit	7471 (30.3)	1973 (21.3)	1147 (58.6)	10,591 (29.5)	30.4
Missing	1377 (5.6)	409 (4.4)	139 (7.1)	139 (7.1)	5.5
*Parity*					
1	10,660 (43.2)	1669 (18.0)	537 (27.5)	12,886 (35.8)	35.4
2	7448 (30.2)	3285 (35.5)	699 (35.7)	11,432 (31.8)	32.1
≥ 2	6369 (25.8)	4248 (45.9)	695 (3535)	11,312 (31.5)	31.6
Missing	208 (0.8)	57 (0.6)	25 (1.3)	290 (0.8)	0.9
*Gravidity*					
< 2	9454 (38.3)	1267 (13.7)	454 (23.2)	11,175 (31.3)	30.6
≥ 2	14,845 (60.1)	7868 (85.0)	1465 (74.9)	24,178 (67.4)	67.7
Missing	386 (1.6)	124 (1.3)	37 (1.9)	547 (1.5)	1.6
*Trimester at booking*					
First	8048 (32.6)	3373 (36.4)	609 (31.1)	12,030 (33.5)	33.1
Second	13,725 (55.6)	4920 (53.1)	1014 (51.8)	19,659 (54.8)	55.1
Third	1619 (6.6)	544 (5.9)	151 (7.7)	2314 (6.5)	6.4
Missing	1293 (5.2)	422 (4.6)	182 (9.3)	1897 (5.3)	5.4

*Eligible for analysis of syphilis screening coverage.

### Coverage of syphilis screening

**Figure 2:**
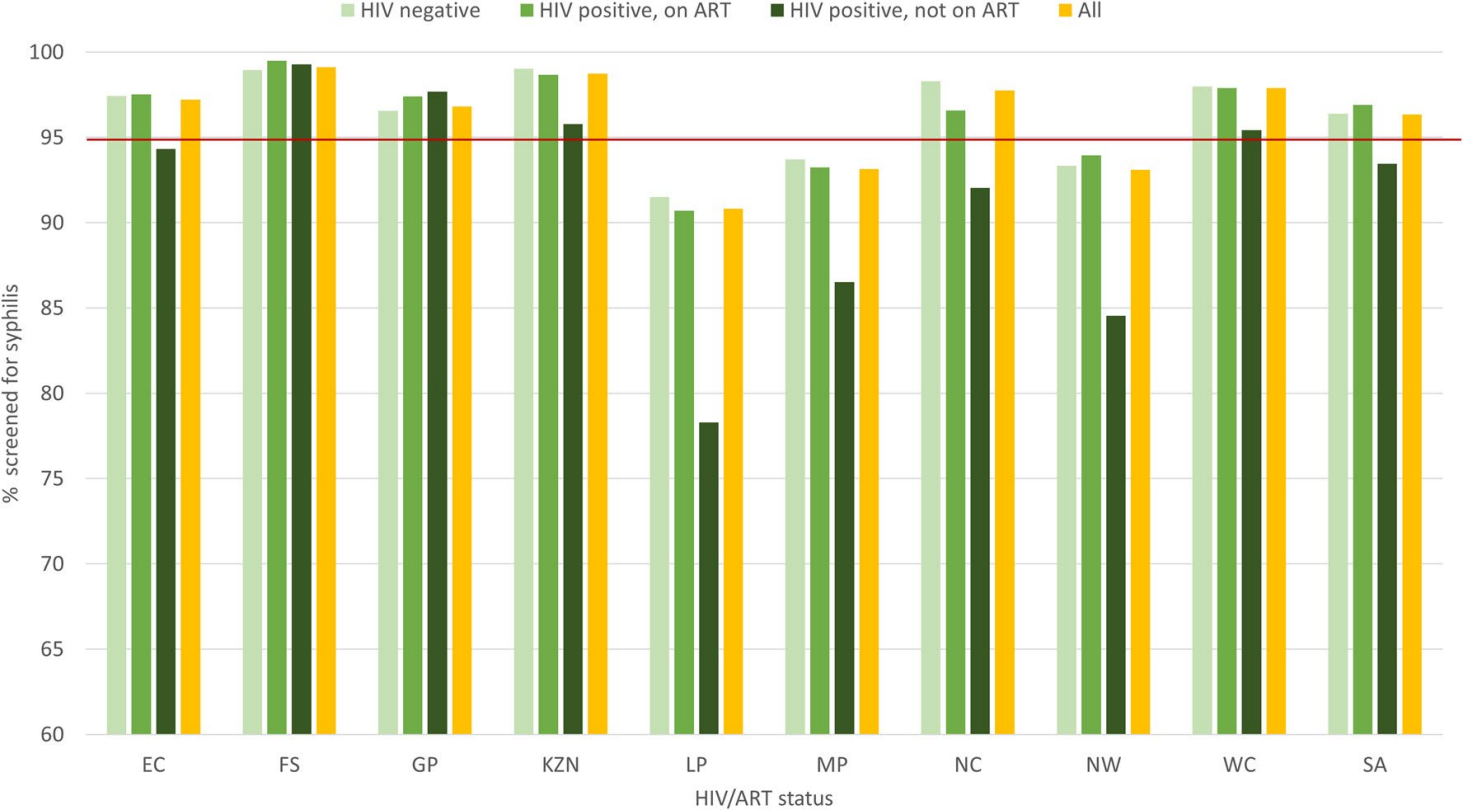
Syphilis screening coverage by province and HIV/ ART status. ART = antiretroviral therapy; Red line = global screening coverage target of 95%; EC = Eastern Cape; FS = Free State; GP = Gauteng; KZN = KwaZulu Natal; LP = Limpopo; MP = Mpumalanga; NC = Northern Cape; NW = North West; WC = Western Cape; SA = South Africa.

The weighted proportion of pregnant women who had syphilis screening done (syphilis screening coverage) was 96.3% [95% CI 95.9–96.7%] overall but varied greatly by province, HIV/ ART status and ANC visit type. The provincial screening coverage ranged from the lowest at 90.8% in Limpopo to the highest at 99.1% in Free State, with three provinces having coverage below the global maternal syphilis screening target of 95%- Limpopo [90.8% (95% CI 88.8–92.5%)], Mpumalanga [93.2% (95% CI 91.1–94.7%)] and North West [93.1% (95% CI 90.8–94.9%)]. Syphilis screening coverage was 97.7% (95% CI 93.9–99.2%) in Northern Cape, the province which had the highest syphilis burden prior to 2015- Fig. 2^11^. The screening coverage was lowest among the HIV positive, not on ART group [93.5% (95% CI 92.2–94.5%)], followed by those who were HIV negative [96.4% (95% CI 95.9–96.8%)] and highest among those who were HIV positive, on ART [96.9% (95% CI 96.5–97.5) Fig. 2. Screening coverage was much lower among those attending ANC for the first time compared to those attending follow up visits—90.2% (95% CI 88.9–91.3%) compared to 99.2 (95% CI 99.0–99.4%). Syphilis screening coverage increased with each gestational trimester at enrolment—92.5% (95% CI 91.4–93.5%), 95.7% (95% CI 95.1–96.1%) and 98.8% (95% CI 96.7–99.0%) among those who enrolled during the first, second and third trimesters. Screening coverage was higher than 95% across all age categories, racial groups, education level and geographical location (rural, peri-urban and rural). Assuming that those with missing screening data were not screened- a calculation which determines the minimum possible screening coverage, the national level screening coverage was 93.1% (95% CI 92.5–93.7%) with similar trends across categories of HIV/ART status, province, age, racial groups, educational level and geographical location.

### Syphilis positivity and care cascade

**Figure 3:**
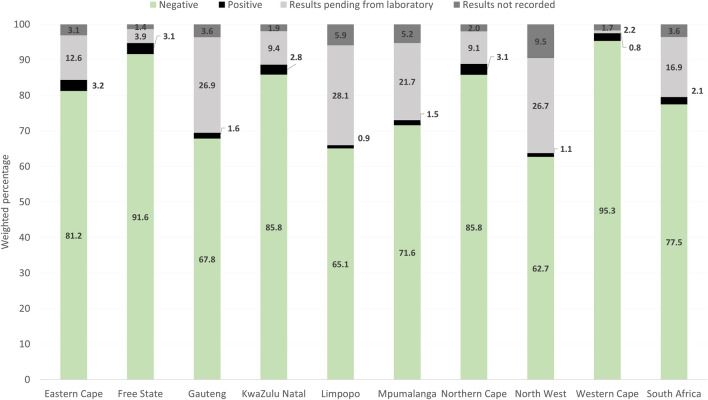
Distribution of syphilis results among pregnant women enrolled in the 2019 ANC sentinel survey by province, N = 34 651.

Of the women who had syphilis screening completed (N = 34 651), 77.5% (95% CI 76.4–78.5%) had results documented as syphilis negative, 16.9% (95% CI 16.0–17.8%) had results pending from the laboratory, 3.6% (95% CI 3.0–4.2%) had results missing and 2.1% (95% CI 1.9–2.3%) were documented as syphilis positive- Fig. 3. Overall, only 79.6% (95% CI 78.5–80.5%) of women screened for syphilis had results documented at the time of enrolment. The proportion who had documented results varied by province, by HIV/ART status and by whether visit was a first visit or a follow up visit. At provincial level, 63.8–97.5% of women screened for syphilis had documented results and in only two provinces—Free State and Western Cape—did this proportion exceed 90%—Fig. 3. Among women who were HIV negative this proportion was 78.7% (95% CI 77.6–79.7%) compared to 85.1% (95% CI 84.0–86.1%) among those HIV positive on ART and 65.4% (95% CI 62.8–67.9%) among those HIV positive, not on ART. Among those attending ANC for the first time, the proportion who had documented results was 45.1% (95% CI 43.0–47.2%) compared to 93.8% (95% CI 93.3–94.3%) attending follow up visits.

**Figure 4:**
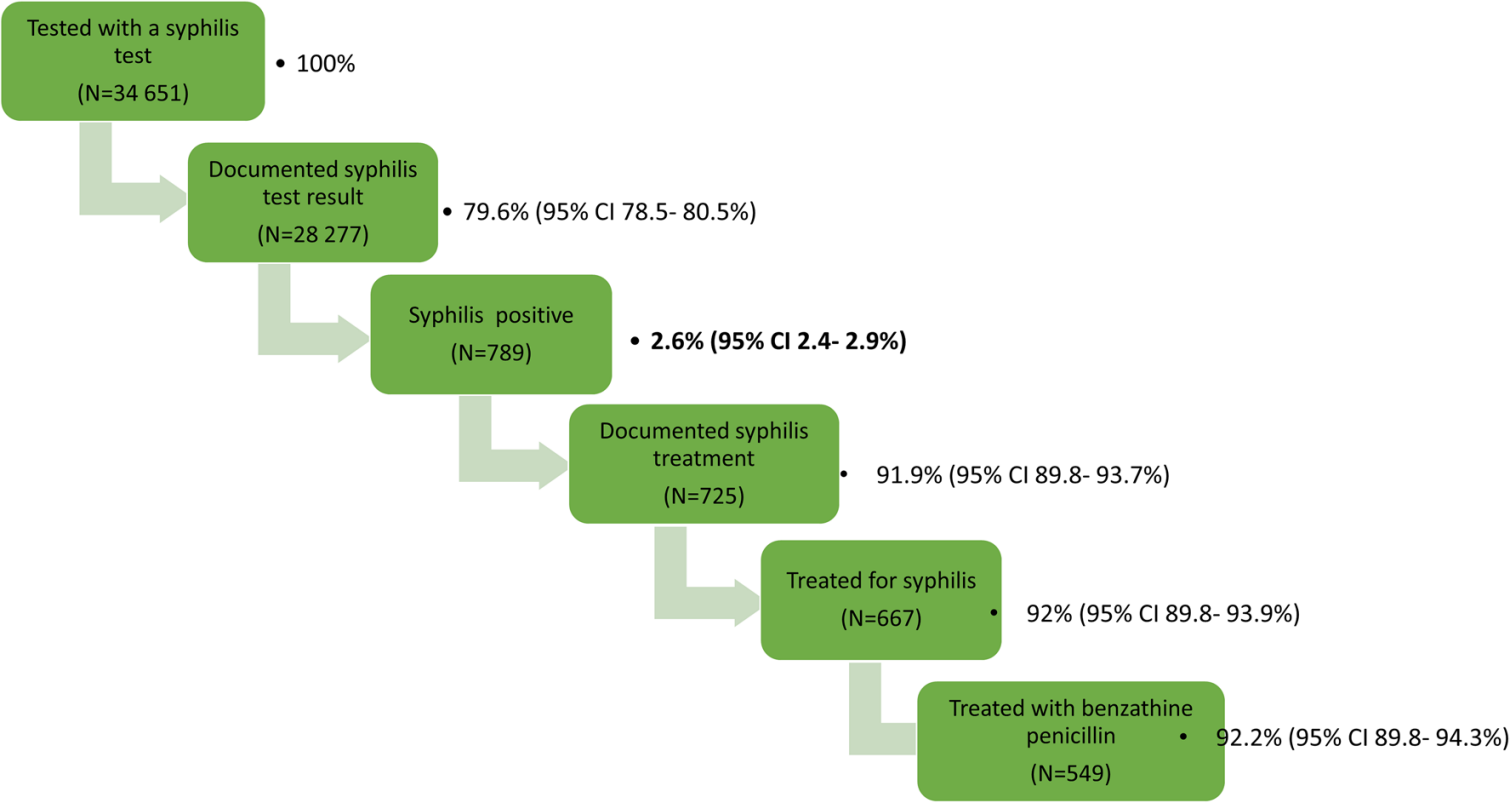
Syphilis screening and treatment cascade among pregnant women enrolled in the 2019 ANC survey, South Africa N = 34 651.

Syphilis positivity among those with documented syphilis screening results was 2.6% (2.4–2.9%). The positivity varied by age, level of education, relationship with partner who fathered the baby, province, location (urban, peri-urban or rural) as well as the maternal HIV/ART status (Table 2). Among those who were syphilis positive, 91.9% (95% CI 89.8–93.7%) had documentation of treatment of whom 92.0% (95% CI 89.8–93.9%) were treated for syphilis, with the majority treated with BPG [92.2% (95% CI 89.8–94.3%)]—Fig. 4.

**Table 2:** Factors associated with being syphilis positive among pregnant women enrolled in the 2019 ANC survey and had documented syphilis screening results, South Africa, N = 24,064*.

Variable	% syphilis positive (95% CI)	Univariable logistic regression OR (95% CI)	*p*-value	Multivariable logistic regression aOR (95% CI)	*p*-value
*Age category (years), n (%)*					
15–19	1.5 (1.2–1.8)	1.00		1.00	
20–24	2.7 (2.4–3.0)	1.84 (1.44–2.36)	< 0.001	1.64 (1.25–2.14)	< 0.001
25–29	2.8 (2.5–3.1)	1.93 (1.50–2.49)	< 0.001	1.43 (1.06–1.91)	0.018
30–34	3.0 (1.6–3.4)	2.06 (1.59–2.67)	< 0.001	1.46 (1.07–2.00)	0.018
35–49	2.6 (2.1–3.3)	1.82 (1.35–2.48)	< 0.001	1.27 (0.89–1.81)	0.188
*Population group*					
Black	2.6 (2.4–2.9)	1.00		1.00	
Mixed race	2.8 (2.4–3.2)	1.06 (0.88–1.27)	0.558	1.31 (1.02–1.69)	0.032
Other^#^	1.1 (0.5–2.3)	0.42 (0.21–0.88)	0.022	0.27 (0.08–0.90)	0.033
*Education*					
None/primary	3.1 (2.6–3.7)	1.58 (1.21–2.08)	0.001	1.59 (1.18–2.16)	0.003
Secondary	2.6 (2.4–2.9)	1.33 (1.07–1.65)	0.009	1.29 (1.02–1.62)	0.032
Tertiary	2.0 (1.6–2.5)	1.00		1.00	
*Relationship with partner who fathered current pregnancy*					
Married	1.8 (1.5–2.3)	1.00		1.00	
Living together as married	2.7 (2.4–3.0)	1.46 (1.16–1.85)	0.001	1.30 (1.04–1.62)	0.020
Not living together but in a relationship	2.8 (2.5–3.0)	1.51 (1.24–1.84)	< 0.001	1.45 (1.17–1.79)	0.001
No relationship	3.4 (2.7–4.3)	1.87 (1.35- 2.59)	< 0.001	1.78 (1.26- 2.51)	0.001
*Age difference with partner who fathered current pregnancy*					
< 5 years	2.7 (2.4–2.9)	1.00		–	–
≥ 5 years	2.6 (2.3–3.0)	0.98 (0.87–1.11)	0.750	–	–
Does not know	2.0 (1.5–2.6)	0.74 (0.55–0.98)	0.038	–	–
*Geo-location*					
Rural	2.2 (1.8–2.6)	1.00		1.00	
Peri-urban	2.8 (2.5–3.1)	1.31 (1.06–1.60)	0.013	1.27 (1.04–1.69)	0.021
Urban	3.1 (2.5–3.7)	1.43 (1.09–1.88)	0.010	1.33 (0.95–1.69)	0.104
*ANC visit*					
Follow up	2.4 (2.2–2.6)	1.00		1.00	
First ANC visit	3.7 (2.9–4.7)	1.59 (1.23–2.06)	< 0.001	1.47 (1.12–1.93)	0.006
*Parity*					
0	2.1 (1.9–2.3)	1.00		1.00	
1	3.0 (2.7–3.4)	1.48 (1.29–1.49)	0.001	0.89 (0.67–1.17)	0.397
≥ 2	2.8 (2.5–3.2)	1.36 (1.18–1.58)	0.001	0.85 (0.62–1.15)	0.295
*Gravidity*					
< 2	1.9 (1.7–2.2)	1.00		1.00	
≥ 2	2.9 (2.7–3.2)	1.55 (1.36–1.78)	< 0.001	1.46 (1.09–1.96)	0.011
*Trimester at booking*					
First	2.7 (2.4–3.0)	1.00			
Second	2.4 (2.2–2.7)	0.90 (0.79–1.02)	0.099		
Third	3.1 (2.5–3.9)	1.18 (0.93–1.50)	0.173		
*HIV/ART status*					
HIV negative	1.9 (1.7–2.1)	1.00		1.00	
HIV positive, on ART	4.2 (3.8–4.7)	2.32 (2.04–2.63)	< 0.001	2.25 (1.91–2.64)	< 0.001
HIV positive, not on ART	4.7 (3.7–5.9)	2.57 (2.03–3.24)	< 0.001	2.24 (1.71–2.93)	< 0.001
*Province*					
Eastern Cape	1.62 (1.08–2.43)	0.021		1.58 (0.96–2.61)	0.070
Free State	1.42 (1.11–1.83)	0.006		1.37 (1.03–1.81)	0.030
Gauteng	1.00			1.00	
KwaZulu- Natal	1.37 (1.09–1.71)	0.006		1.46 (1.12–1.89)	0.005
Limpopo	0.57 (0.40–0.82)	0.002		0.88 (0.57–1.35)	0.566
Mpumalanga	0.85 (0.50–1.44)	0.545		0.96 (0.56–1.67)	0.891
North West	0.72 (0.48–1.09)	0.123		0.91 (0.57–1.46)	0.700
Northern Cape	1.50 (1.11–2.02)	0.008		1.45 (1.01–2.07)	0.044
Western Cape	0.94 (0.75–1.19)	0.633		0.94 (0.71–1.25)	0.681

*This is 24 064/28 277 (85.1%) for whom syphilis results were available; # other race includes white, Asian and any other race individuals self-identified as; *OR* Odds ratio, *aOR* adjusted odds ratio, *CI* Confidence interval OR (95% CI).

### Factors associated with maternal syphilis positivity

In univariable analyses, maternal syphilis positivity increased with each increasing age category up to age 30–34 years compared to age 15–19 years. Maternal syphilis positivity was higher among women with primary or secondary education compared to tertiary education, among women not married to the partners who fathered the current pregnancy compared to those married and among women in peri-urban and urban settings compared to rural settings. Maternal syphilis positivity was highest among women enrolled in Eastern Cape, Free State.

Documented syphilis treatment refers to women who had information on whether treatment was received or not while treated for syphilis refers to women documented as having received the treatment.

KwaZulu-Natal and Northern Cape compared to Gauteng—the designated reference province because of its syphilis prevalence which was closest to the national average. Maternal syphilis positivity was also higher among those attending ANC for the first time, those with a higher gravidity (≥ 2) compared to lower and those who were HIV positive compared to those who were HIV negative regardless of ART status (Table 2).

In a multivariable model adjusting for the effect of age, racial group, maternal education, relationship status, province, location, visit type, gravidity, parity and HIV/ART status—women living with HIV had a greater likelihood of being syphilis positive compared to those not living with HIV regardless of ART status—aOR 2.25 (95% 1.91–2.64) among those on ART and aOR 2.24 (95% CI 1.72–2.93) among those not on ART. Compared to the 15–19 year age group, being 20–24 years old was associated with the greatest likelihood of being positive for syphilis [aOR 1.64 (95% CI 1.25–2.14)] followed by being 30–34 years old [aOR 1.46 (95% CI 1.07–2.00)] and being 25–29 years old [aOR 1.43 (95% CI 1.06–1.91)]. Being of white, Indian, Asian or any other ethnicity was associated with lower likelihood of being syphilis seropositive [aOR 0.27 (95% CI 0.08–0.90) compared to being of black African ethnicity. Maternal education was independently associated with syphilis seropositivity, with greater likelihood among women with no education or those that had not completed primary school education [aOR 1.59 (95% CI 1.18–2.16)] compared to those who had completed tertiary education. Maternal relationship with partner was also independently associated with being syphilis seropositive with the greatest likelihood among women who had no relationship with the partners who fathered their children [aOR 1.78 (95% CI 1.26–2.51)] compared to those who were married to their partners. Pregnant women enrolled from facilities in peri-urban areas were independently more likely to be syphilis seropositive compared to those enrolled from facilities in rural areas [aOR 1.27 (95% CI 1.04–1.69)]. Women attending ANC for the first time were more likely to be syphilis seropositive compared to those attending follow up visits [aOR 1.47 (95% CI 1.12–1.93)], as were those with higher gravidity (≥ 2)—[aOR 1.46 (95% CI 1.08–1.96). Across three provinces, syphilis seropositivity was 1.37–1.46 times higher than Gauteng the reference province.

In a multivariable model that allowed for interaction between province and HIV/ART status, for seven provinces -Free State, Gauteng, KwaZulu-Natal, Limpopo, Mpumalanga, Northern Cape and North West—HIV positive women on ART were 1.98–8.92 times more likely to be syphilis positive compared to HIV negative women in the same province. On the other hand, HIV positive women not on ART were 1.77–9.76 times more likely to be syphilis positive compared to those who were HIV negative in five provinces—Eastern Cape, Gauteng, KwaZulu-Natal, Limpopo and North West. HIV negative women from all provinces except Eastern Cape, Limpopo, Mpumalanga and North West had 1.75–2.21 times greater likelihood of being syphilis positive compared to HIV negative women in Gauteng. HIV positive women on ART from all provinces except the Western Cape were just as likely as those from Gauteng to be syphilis positive. Among those HIV positive but not on ART, women from all provinces except Free State were as likely as those from Gauteng to be syphilis positive. (Supplementary Table S1).The model with the interaction term performed better compared to the one without [area under the curve (UAC) 0.67 (95% CI 0.65–0.69) vs. 0.66 (95% CI 0.64–0.68), *p* = 0.009]. In an analysis restricted to women attending a follow up visit at enrolment: being HIV positive on ART—aOR 3.76 (95% CI 2.32–6.08) and being HIV positive but not on ART—aOR 3.65 (95% CI 1.56–8.52) remained independently associated with syphilis positivity compared to being HIV negative in the reference province (Gauteng)—supplementary Table S2. Similar better model fit was observed in the model including an interaction term UAC 0.685 (95% CI 0.662–0.777) versus 0.677 (95% CI 0.654–0.700), *p* = 0.022.

## Discussion

In this analysis of syphilis data from the 2019 ANC survey, we found a national syphilis screening coverage of 96.4% among eligible pregnant women. The proportion of women screened for syphilis was lowest among HIV positive pregnant women not on ART and highest among those who were HIV positive on ART. Among those screened only 79.6% had documented results with 2.6% being syphilis positive. Coverage of treatment with BPG was below the global target for treatment (95%) among those who tested syphilis positive. Syphilis seropositivity was associated with living with HIV regardless of ART use, province, maternal age, maternal education, relationship with father of unborn child and first ANC visit attendance at enrolment.

The syphilis screening coverage at 96.4% showed that South Africa had attained the first 95 of the syphilis care cascade and was similar to the 96.1% estimated during the 2017 ANC survey^10^. However, this proportion would be below 95% if women who had missing syphilis screening data were assumed to be unscreened^1^. Of concern was the high proportion of women (almost 20%) who were documented as screening done but had results pending from the laboratory or not recorded in their medical records for whatever reason. The introduction of nationally approved and validated rapid syphilis screening tests will likely reduce the turn-around time for results and allow attending health care workers to respond quickly to positive results. The syphilis screening coverage which was lowest among those who were HIV positive but not on ART could have been a result of the women being newly diagnosed as HIV positive by onsite rapid HIV testing but not had syphilis screening done or documented as it mostly done through centralized laboratory testing. Introduction of dual HIV/ syphilis tests for women previously HIV negative or for whom HIV status is unknown will prevent this differential coverage of syphilis screening by HIV status. Dual HIV/ syphilis testing has been found to be cost-effective in South Africa^13^ and the country plans to introduce a dual HIV/ syphilis screening test, subject to development of a testing algorithm and a quality assurance programme. Because dual HIV/ syphilis tests cannot be used by known HIV positive on ART, planning for introduction of the dual HIV/ syphilis testing will require the country to take into the consideration provincial level differences in HIV prevalence, ART coverage and likely yield of syphilis screening.

The global plan for the elimination of MTCTs requires that 95% of syphilis positive women receive treatment with at least one dose of BPG at least 30 days before delivery^1^. While the full evaluation of the performance of this indicator was not possible because data on date of maternal treatment and expected date of delivery were not collected, only 92% of syphilis positive women had syphilis treatment documented with a further 92% treated with BPG. Given that about 55% were in their second trimester at the time of booking and syphilis can cause preterm delivery, ensuring that syphilis positive women are treated as soon as possible is essential for the prevention of adverse pregnancy events. Introduction of rapid tests and a steady supply of BPG will facilitate same day treatment of women who are syphilis positive. In the face of BPG stock outs, the country has prioritised pregnant women for treatment with the drug and has requested emergency use authorizations for formulations, which are not registered by the country’s medicines regulator^14^. The process of obtaining these authorisations for individual women involves obtaining consent and completing some paperwork, processes that can result in delays in starting treatment.

Like previous ANC surveys in South Africa, the 2019 survey found that syphilis prevalence varied with age, province, location (rural, urban, and peri-urban) and HIV status. National surveys from Cameroon and Rwanda found an association between syphilis seropositivity with location and age^15,16^. Studies from Ethiopia found that syphilis prevalence among HIV positive pregnant women was up to 14 times higher than that among those who were HIV negative but did not stratify by ART status^17,18^. The stratification by ART status in the analysis for factors associated with syphilis seropositivity was new for South Africa although it has been reported in studies among pregnant women and other populations from elsewhere^19–21^. In a study of pregnant women from China, being on ART was associated with greater likelihood of syphilis infection in adjusted analyses^20^. We found a higher likelihood of being syphilis positive among those on ART compared to HIV negatives women in some provinces and not in others. Syphilis is thought to facilitate HIV acquisition among those who are HIV negative^22^, while HIV infection is associated with repeat episodes of syphilis and a tendency to remain positive after treatment—that is being serofast on non-treponemal assays^23^. The mechanisms by which HIV facilitates repeat syphilis infection is not fully understood and may be biological or behavioural^23,24^. Therefore, syphilis screening should be offered to all pregnant women regardless of HIV or ART treatment status. Our survey also found that the more casual the maternal relationship with the partner who fathered current pregnancy was, the greater the likelihood of the mother being syphilis positive. This finding was similar to that from a studies from the Demographic Republic of Congo, Cameroon and Rwanda which found higher syphilis seroprevalence among unmarried pregnant women compared to married^15,16,25^. This finding highlights the need to strengthen primary and secondary syphilis prevention efforts such as promoting condom use among both HIV positive and HIV negative women, male medical circumcision, reducing multiple and concurrent sexual partnerships as well as partner notification and treatment in order to reduce syphilis incidence among men and women of reproductive age.

Our study presented data on syphilis positivity from a large nationally representative sample. The data used was collected from women attending both first and follow up visits, providing data on the frequency of the disease throughout pregnancy. However, our study had some limitations. The study excluded women who did not have HIV results largely because their blood specimen was haemolysed or had no valid HIV test results. To overcome this limitation facilities were requested to continue enrolling until the end of the survey as opposed to their target sample size in order to make up for hemolysed samples. Even with the inflated enrolment, the analysis of syphilis coverage and seropositivity included 86% and 83% of the total enrolled respectively. The women excluded and those included in these analyses may differ in ways that could affect syphilis screening or seropositivity leading to selection bias. Data on syphilis testing coverage and test results were obtained from medical record review and abstraction. This excluded women who had specimens collected around the day of enrolment as results were still pending from the laboratory. The data collection form did not collect information on the type (treponemal or non-treponemal) or name of serological tests used for syphilis screening. Midwives may have abstracted data on results of Treponemal tests used for example in rapid tests. These tests remain positive in people with past infection and when positive need to be confirmed with a non-treponemal test in the laboratory in order to exclude past infection. The sole use of specific tests for screening may overestimate syphilis positivity. South Africa had no nationally validated rapid syphilis-testing algorithm at the time of the survey, but anecdotal reports suggested the Free State and Western Cape provinces used rapid syphilis testing in most districts. Everywhere else, centralised laboratory testing was the main syphilis screening and testing modality. Parallel testing of specimens collected in the survey would have established more accurate estimates of syphilis positivity and evaluated the performance of the record review in the estimation. Lastly, the study was conducted in late 2019, prior to the onset of the COVID-19 pandemic. Early studies study of impact of COVID-19 on ANC attendance found some minimal decrease in attendance during the periods of lockdown restrictions^26,27^. Despite these limitations, the study has generated data, which is essential for monitoring and evaluating key prevention of MTCTs interventions in the country.

In conclusion, there was high syphilis screening coverage and high treatment coverage although the coverage of treatment failed to meet the global targets for the syphilis care cascade. In most provinces, syphilis positivity was higher among HIV positive women regardless of ART status. There is need to strengthen screening for syphilis among the HIV negative and those not on ART strengthening the case for the introduction of rapid dual HIV/syphilis testing in the country. Since initiation of appropriate treatment was sub-optimal, it needs to be remediated to prevent MTCTs. Lastly future surveys could document dates of syphilis treatment and expected dates of delivery, information useful for evaluating adequacy of syphilis treatment.

## Methods

### Setting

South Africa has conducted ANC HIV prevalence surveys since 1990. The surveys were designed to monitor trends in HIV prevalence among pregnant women at national, provincial and district levels, and were conducted annually until 2015 and every two years since then. The surveys enroll consenting pregnant women aged 15–49 years attending ANC at public health clinics participating as sentinel sites. Syphilis prevalence was measured in the surveys annually until 2011, changing to every four years since then. Until 2014, only women attending ANC for the first time (booking visit) regardless of HIV status were enrolled with enrollments opening up to all women attending ANC regardless of the type of visit after 2014. ANC attendance for at least one visit is high in the country and was estimated at 94% in 2016 with 76% of women attending at least four visits^28^. In 2019, 1589 out of about 4000 primary care clinics providing ANC services participated in the survey.

Syphilis testing in South African antenatal clinics is largely accomplished through centralized laboratory testing at the National Health Laboratory Services (NHLS) laboratories. As of January 2022, the NHLS runs a network of at least 260 public health laboratories in all provinces in the country. Blood specimens are collected from women attending care and sent to the closest local or regional laboratories based in district and regional hospitals using a courier service. At the laboratory, they test for syphilis using either the traditional or the reverse testing algorithms^29^. With the reverse testing algorithm, the laboratory screens the serum specimen using a specific treponemal test (TPAb) and confirms the treponemal infection using the rapid plasma reagin test (RPR) both qualitative and quantitative^29^. On the other hand, with the traditional testing algorithm, the laboratory screens the serum specimen using a non-specific test—rapid plasma reagin test (RPR) and confirms with a specific treponemal test (TPAb)^29^. The choice of traditional versus reverse testing algorithm is made by the responsible pathologist at the local laboratory. A few provinces and districts in the country have also implemented rapid syphilis testing where screening with a treponemal test happens on site [Personal communication, NDOH STI Manager] and when positive, a blood specimen is collected and sent to a central laboratory for confirmatory testing with an RPR. At the time of the 2019 ANC survey, there was no nationally validated or recommended algorithm for rapid syphilis testing in the country.

### Design

The 2019 edition of the South African ANC survey was cross-sectional and employed a stratified cluster sampling design to sample pregnant women aged 15–49 years^30^. As in the previous surveys, the sample size determination was guided by two main objectives: i) to estimate HIV prevalence within an acceptable level of precision, and ii) to measure change in HIV prevalence over time. The sample size calculation was performed to estimate HIV prevalence at district level with a precision level of 3–5%, with 95% confidence interval (CI), design effect of 1.5 and 10% error rate to account for loss of specimens, data collection forms or incomplete reporting. The planned sample size for the 2019 survey was 36 000 women.

### Data collection

The survey was conducted from 1 October to 15 November 2019. Details of the objectives, sampling and data collection methods have been previously described^30^. Briefly, data collection procedures included written informed consent, a brief interview, medical record review and blood specimen collection. Pregnant women aged 15–49 years who were able to give consent were offered enrolment into the survey during ANC visits. Willing and eligible women provided written informed consent to participate in the survey after which a nurse-administered interview and maternity record review with abstraction of relevant data were conducted to collect information on key maternal variables. These variables included location, date of specimen collection, age of the woman and father of unborn child, race of woman, nature of relationship with father of unborn child (married, living as married, single), antenatal visit type, gestational age at enrolment, gestational age at first booking, gravidity, parity, HIV status, ART initiation, timing of ART initiation, routine maternal syphilis screening, syphilis test results and syphilis treatment.

### Laboratory procedures

Completed data collection forms and specimens were sent to designated regional serology laboratories where specimens were processed, and HIV serology testing conducted. Two fourth-generation HIV-1 enzyme immunoassays (EIA) were used to test for HIV infection, following the manufacturer’s instructions—including appropriate quality control specimens. The brand of screening (EIA 1) and confirmatory (EIA 2) tests differed by regional laboratory—Supplementary Table S3. Specimens that were non-reactive on first EIA were classified as HIV seronegative. All samples that were reactive using EIA 1 were re-tested using a second and different EIA (EIA 2). If EIA 1 and EIA 2 agreed, the result was classified “HIV-positive”. If EIA 1 and EIA 2 did not agree, the result was recorded as “discrepant”. The specimen information, including EIA 1 and EIA 2 results, were captured in an electronic lab information system called TrakCare. ART treatment status was determined from record review.

### Data management

At the reference laboratory, data clerks scanned data collection forms into an electronic database using optical mark recognition (OMR) software. The electronic database was uploaded on the National Institute for Communicable Diseases (NICD) server and data exported directly to STATA® version 14.2 [Stata Corporation, College Station, Texas, United States] for data cleaning and analysis. All EIA screening and confirmatory test results were downloaded from TrakCare (the NHLS lab electronic laboratory information system) as Excel® files. The Excel spreadsheets were also exported into STATA® version 14.2 for merging with data from study forms. The final database excluded observations for participants outside the age range of 15–49 years, those with no interview data, rejected or lost specimens and those with equivocal or unconfirmed HIV test results and those who had missing syphilis testing data.

### Data analysis

Data analysis accounted for the survey design (clustering within primary sampling units [PSUs]—clinics and stratification by district) and was weighted for sample size realization (at district level) and for the Statistics South Africa 2019 mid-year population estimate for women of reproductive age (15–49 years) at provincial level. Because sites were sampled using probability proportional to size method, and that the sampling period was fixed, this provided a self-weighted sample at district level. A population finite correction factor was added, to adjust for the > 5% of PSUs sampled without replacement from a finite population of about 4 000 public facilities. Descriptive analyses included data distribution nationally and by age, province, location (rural, peri-urban and rural), relationship with partner who fathered current pregnancy, race group, parity, gravidity, visit type (first or follow-up ANC visit) and HIV treatment status (HIV positive on ART at booking, HIV positive not on ART at booking and HIV negative). Median and interquartile ranges (IQR) were reported for continuous variables, while frequencies were reported for categorical variables. The outcomes for these analyses were i) coverage of syphilis screening—defined as proportion of pregnant women with a documented syphilis test, ii) syphilis positivity—defined as the proportion of pregnant women who had syphilis positive result according to national testing practice iii) documented coverage of any syphilis treatment and iv) documented coverage of treatment with one or more doses of BPG. Univariable and multivariable logistic regression was used to determine factors associated with being syphilis positive adjusting for HIV treatment status and other confounding factors. Variables with *p*-values < 0.05 in univariable analysis were included in the multivariable model with age, racial group, province and HIV/ART status included a priori. HIV/ ART status was determined from a combination of HIV status determined in the laboratory (HIV positive vs HIV negative) and from ART status at booking for those who were HIV positive. As syphilis positivity in the country was known to vary with both HIV status and province and HIV prevalence and coverage of treatment was also known to vary by province, an additional multivariable model which allowed for interaction between province of enrolment and HIV/ART status was also fit. Both models only included women with complete data. Model performance in predicting syphilis positivity as determined by receiver operator curves was used to compare the model with an interaction term included to the one without the interaction term. The model with a statistically significant larger area under the curve was considered superior and reported on. A sub-group analysis including women attending follow up visits and therefore likely to have all testing completed and included was conducted to check the robustness of the estimates. All analyses were conducted using STATA® version 14.2.

### Ethical considerations

Participation in the survey was voluntary, requiring written informed consent. Participants could withdraw from the study at any time and this withdrawal did not influence the care received as part of routine antenatal care. Participants were not compensated for their participation. To protect the confidentiality of participants’ information, the data collection form was submitted without patient identification. Ethical clearance and approval was obtained from the University of the Witwatersrand Human Research Ethics Committee (Medical) and from each of the nine provincial health research ethics committees. The study protocol was reviewed in accordance with the US Centers for Disease Control and Prevention (CDC) human research protection procedures and was determined to be research, but CDC investigators did not interact with human subjects or have access to identifiable data or specimens for research purposes.

## Supplementary Information

Supplementary Information.

## Data Availability

The data that support the findings of this study are available from National Department of Health, South Africa but restrictions apply to the availability of these data, which were collected on behalf of the National Department of Health, South Africa for the current study, and so are not publicly available. Data are however available from the corresponding author Tendesayi Kufa (tendesayikc@nicd.ac.za) upon reasonable request and with permission of National Department of Health, South Africa.
