# Supplementary material for: Syphilis screening coverage and positivity by HIV treatment status among South African pregnant women enrolled in the 2019 antenatal HIV sentinel survey
Source: Sci Rep. 2023 Apr 1;13:5322. doi: 10.1038/s41598-023-32456-0 (PMC10067819; doi:10.1038/s41598-023-32456-0)
Supplement: Supplementary file 1 — Supplementary Information. [file 41598_2023_32456_MOESM1_ESM.docx]

**Supplementary Table S1: Factors associated with being syphilis positive among pregnant women enrolled in the 2019 ANC survey and had documented syphilis screening results in a model including interaction, South Africa, N=** **24,038**

| **Variable** | **% syphilis positive**  **(95% CI)** | **Univariable logistic regression**  **OR (95% CI)** | **p-value** | **Multivariable logistic regression**  **aOR (95% CI)** | **p-value** |
| --- | --- | --- | --- | --- | --- |
| *Age category (years), n (%)* |  |  |  |  |  |
| 15-19 | 1.5 (1.2- 1.8) | 1.00 |  | 1.00 |  |
| 20- 24 | 2.7 (2.4- 3.0) | 1.84 (1.44- 2.36) | <0.001 | 1.65 (1.26- 2.17) | <0.001 |
| 25- 29 | 2.8 (2.5- 3.1) | 1.93 (1.50- 2.49) | <0.001 | 1.45 (1.08- 1.94) | 0.014 |
| 30- 34 | 3.0 (1.6- 3.4) | 2.06 (1.59- 2.67) | <0.001 | 1.49 (1.09- 2.03) | 0.019 |
| 35- 49 | 2.6 (2.1- 3.3) | 1.82 (1.35- 2.48) | <0.001 | 1.28 (0.90- 1.84) | 0.173 |
| *Population group* |  |  |  |  |  |
| Black | 2.6 (2.4- 2.9) | 1.00 |  | 1.00 |  |
| Mixed race | 2.8 (2.4- 3.2) | 1.06 (0.88- 1.27) | 0.558 | 1.18 (0.91- 1.53) | 0.200 |
| Other^#^ | 1.1 (0.5- 2.3) | 0.42 (0.21- 0.88) | 0.022 | 0.26 (0.08- 0.88) | 0.031 |
| *Education* |  |  |  |  |  |
| None/primary | 3.1 (2.6- 3.7) | 1.58 (1.21- 2.08) | 0.001 | 1.57 (1.17- 2.15) | 0.003 |
| Secondary | 2.6 (2.4- 2.9) | 1.33 (1.07- 1.65) | 0.009 | 1.28 (1.02- 1.61) | 0.039 |
| Tertiary | 2.0 (1.6- 2.5) | 1.00 |  | 1.00 |  |
| *Relationship with partner who fathered current pregnancy* |  |  |  |  |  |
| Married | 1.8 (1.5- 2.3) | 1.00 |  | 1.00 |  |
| Living together as married | 2.7 (2.4- 3.0) | 1.46 (1.16- 1.85) | 0.001 | 1.30 (1.04- 1.63) | 0.019 |
| Not living together but in a relationship | 2.8 (2.5- 3.0) | 1.51 (1.24- 1.84) | <0.001 | 1.45 (1.17- 1.79) | 0.001 |
| No relationship | 3.4 (2.7- 4.3) | 1.87 (1.35- 2.59) | <0.001 | 1.78 (1.26- 2.51) | 0.001 |
| *Age difference with partner who fathered current pregnancy* |  |  |  |  |  |
| <5 years | 2.7 (2.4- 2.9) | 1.00 |  | -- | -- |
| ≥5 years | 2.6 (2.3- 3.0) | 0.98 (0.87- 1.11) | 0.750 | -- | -- |
| Does not know | 2.0 (1.5- 2.6) | 0.74 (0.55- 0.98) | 0.038 | -- | -- |
| *Geo-location* |  |  |  |  |  |
| Rural | 2.2 (1.8- 2.6) | 1.00 |  | 1.00 |  |
| Peri-urban | 2.8 (2.5- 3.1) | 1.31 (1.06- 1.60) | 0.013 | 1.26 (0.95- 1.68) | 0.109 |
| Urban | 3.1 (2.5- 3.7) | 1.43 (1.09- 1.88) | 0.010 | 1.34 (1.05- 1.70) | 0.018 |
| *ANC visit* |  |  |  |  |  |
| Follow up | 2.4 (2.2- 2.6) | 1.00 |  | 1.00 |  |
| First ANC visit | 3.7 (2.9- 4.7) | 1.59 (1.23- 2.06) | <0.001 | 1.47 (1.12- 1.93) | 0.005 |
| *Parity* |  |  |  |  |  |
| 0 | 2.1 (1.9- 2.3) | 1.00 |  | 1.00 |  |
| 1 | 3.0 (2.7- 3.4) | 1.48 (1.29- 1.49) | 0.001 | 0.89 (0.67- 1.17) | 0.397 |
| ≥2 | 2.8 (2.5- 3.2) | 1.36 (1.18- 1.58) | 0.001 | 0.85 (0.62- 1.15) | 0.295 |
| *Gravidity* |  |  |  |  |  |
| <2 | 1.9 (1.7- 2.2) | 1.00 |  | 1.00 |  |
| ≥2 | 2.9 (2.7- 3.2) | 1.55 (1.36- 1.78) | <0.001 | 1.45 (1.08- 1.95) | 0.013 |
| *Trimester at booking* |  |  |  |  |  |
| First | 2.7 (2.4- 3.0) | 1.00 |  |  |  |
| Second | 2.4 (2.2- 2.7) | 0.90 (0.79- 1.02) | 0.099 |  |  |
| Third | 3.1 (2.5- 3.9) | 1.18 (0.93- 1.50) | 0.173 |  |  |
| *HIV/ART status by province* |  |  |  |  |  |
| *Eastern Cape* |  |  |  |  |  |
| HIV negative | 3.0 (1.9- 4.7) | 1.00 |  | 1.00 |  |
| HIV positive , on ART | 4.1 (3.1- 5.5) | 1.38 (1.01- 1.89) | 0.046 | 1.39 (0.96- 1.99) | 0.080 |
| HIV positive , not on ART | 9.2 (5.8- 14.1) | 3.24 (2.23- 4.70) | <0.001 | 2.87 (1.91- 4.34) | <0.001 |
| *Free State* |  |  |  |  |  |
| HIV negative | 2.7 (2.1- 3.4) | 1.00 |  | 1.00 |  |
| HIV positive , on ART | 5.1 (4.0- 6.5) | 1.98 (1.40- 2.80) | <0.001 | 1.98 (1.38- 2.84) | <0.001 |
| HIV positive , not on ART | 1.6 (0.7- 4.1) | 0.61 (0.25- 1.52) | 0.290 | 0.30 (0.08- 1.16) | 0.080 |
| *Gauteng* |  |  |  |  |  |
| HIV negative | 1.6 (1.3- 2.0) | 1.00 |  | 1.00 |  |
| HIV positive , on ART | 4.1 (3.3- 5.0) | 2.58 (1.91- 3.50) | <0.001 | 2.89 (1.98- 4.22) | <0.001 |
| HIV positive , not on ART | 4.0 (2.4- 6.8) | 2.55 (1.42- 4.60) | 0.002 | 3.40 (1.77- 6.55) | <0.001 |
| *KwaZulu Natal* |  |  |  |  |  |
| HIV negative | 2.0 (1.6- 2.5) | 1.00 |  | 1.00 |  |
| HIV positive , on ART | 4.8 (4.2- 5.6) | 2.46 (1.93- 3.14) | <0.001 | 2.20 (1.71- 2.83) | <0.001 |
| HIV positive , not on ART | 4.6 (3.0- 7.1) | 2.33 (1.43- 3.80) | 0.001 | 1.77 (1.03- 3.04) | 0.037 |
| *Limpopo* |  |  |  |  |  |
| HIV negative | 0.5 (0.3- 0.9) | 1.00 |  | 1.00 |  |
| HIV positive , on ART | 4.7 (3.1- 6.9) | 9.70 (4.66- 20.21) | <0.001 | 8.92 (4.02- 19.79) | <0.001 |
| HIV positive , not on ART | 4.2 (1.4- 11.8) | 8.68 (2.44- 30.91) | <0.001 | 9.76 (2.62- 36.45) | 0.001 |
| *Mpumalanga* |  |  |  |  |  |
| HIV negative | 1.2 (0.8- 1.8) | 1.00 |  | 1.00 |  |
| HIV positive , on ART | 3.5 (2.0- 6.2) | 3.13 (1.83- 5.34) | <0.001 | 3.16 (1.60- 6.23) | 0.001 |
| HIV positive , not on ART | 3.6 (1.2- 10.8) | 3.21 (1.14- 8.99) | 0.027 | 2.38 (0.80- 7.35) | 0.132 |
| *Northern Cape* |  |  |  |  |  |
| HIV negative | 3.0 (2.3- 3.9) | 1.00 |  | 1.00 |  |
| HIV positive , on ART | 5.7 (3.8- 8.6) | 1.96 (1.20- 3.20) | 0.007 | 1.92 (1.12- 3.30) | 0.018 |
| HIV positive , not on ART | 4.1 (1.4- 11.5) | 1.37 (0.48- 4.12) | 0.572 | 1.43 (0.49- 4.22) | 0.515 |
| *North West* |  |  |  |  |  |
| HIV negative | 1.4 (0.8- 2.2) | 1.00 |  | 1.00 |  |
| HIV positive , on ART | 2.9 (1.8- 4.5) | 2.16 (1.17- 3.98) | 0.014 | 2.22 (1.10- 4.49) | 0.026 |
| HIV positive , not on ART | 2.9 (0.4- 15.2) | 2.55 (1.42- 4.60) | 0.020 | 3.40 (1.77- 6.55) | <0.001 |
| *Western Cape* |  |  |  |  |  |
| HIV negative | 2.1 (1.8- 2.5) | 1.00 |  | 1.00 |  |
| HIV positive , on ART | 2.4 (1.7- 3.5) | 1.16 (0.78- 1.70) | 0.477 | 1.40 (0.90- 2.17) | 0.138 |
| HIV positive , not on ART | 3.9 (2.2- 6.7) | 1.88 (1.03- 3.41) | 0.039 | 1.52 (0.74- 3.12) | 0.215 |
| *HIV negative* |  |  |  |  |  |
| Gauteng | 1.6 (1.3- 2.0) | 1.00 |  | 1.00 |  |
| Eastern Cape | 3.0 (1.9- 4.7) | 1.90 (1.14- 3.16) | 0.013 | 2.21 (1.18- 4.14) | 0.013 |
| Free State | 2.7 (2.1- 3.4) | 1.67 (1.18- 2.34) | <0.001 | 1.82 (1.23- 2.73) | 0.003 |
| KwaZulu-Natal | 2.0 (1.6- 2.5) | 1.26 (0.92- 1.73) | 0.154 | 1.75 (1.18- 2.58) | 0.005 |
| Limpopo | 0.5 (0.3- 0.9) | 0.31 (0.16- 0.58) | <0.001 | 0.48 (0.23- 0.99) | 0.048 |
| Mpumalanga | 1.2 (0.8- 1.8) | 0.71 (0.44- 1.16) | 0.172 | 0.93 (0.54- 1.60) | 0.793 |
| Northern Cape | 3.0 (2.3- 3.9) | 1.89 (1.32- 2.72) | 0.001 | 1.91 (1.21- 3.02) | 0.005 |
| North West | 1.4 (0.8- 2.2) | 0.83 (0.48- 1.44) | 0.517 | 1.11 (0.58- 2.19) | 0.749 |
| Western Cape | 2.1 (1.8- 2.5) | 1.31 (0.98- 1.76) | 0.068 | 1.33 (0.90- 1.94) | 0.150 |
| *HIV positive on ART* |  |  |  |  |  |
| Gauteng | 4.1 (3.3- 5.0) | 1.00 |  | 1.00 |  |
| Eastern Cape | 4.1 (3.1- 5.5) | 1.01 (0.70- 1.47) | 0.945 | 1.06 (0.70- 1.62) | 0.787 |
| Free State | 5.1 (4.0- 6.5) | 1.28 (0.91- 1.78) | 0.155 | 1.25 (0.87- 1.80) | 0.225 |
| KwaZulu Natal | 4.8 (4.2- 5.6) | 1.20 (0.92- 1.58) | 0.184 | 1.33 (0.98- 1.80) | 0.067 |
| Limpopo | 4.7 (3.1- 6.9) | 1.15 (0.72- 1.84) | 0.552 | 1.49 (0.87- 2.55) | 0.148 |
| Mpumalanga | 3.5 (2.0- 6.2) | 0.87 (0.46- 1.63) | 0.654 | 1.01 (0.50- 2.07) | 0.968 |
| Northern Cape | 5.7 (3.8- 8.6) | 1.44 (0.89- 2.33) | 0.139 | 1.27 (0.75- 2.15) | 0.371 |
| North West | 2.9 (1.8- 4.5) | 0.70 (0.42- 1.16) | 0.168 | 0.85 (0.49- 1.48) | 0.578 |
| Western Cape | 2.4 (1.7- 3.5) | 0.59 (0.38- 0.90) | 0.015 | 0.64 (0.41- 0.99) | 0.045 |
| *HIV positive not on ART* |  |  |  |  |  |
| Gauteng | 4.0 (2.4- 6.8) | 1.00 |  | 1.00 |  |
| Eastern Cape | 9.2 (5.8- 14.1) | 2.41 (1.51- 5.04) | 0.019 | 1.87 (0.80- 4.40) | 0.151 |
| Free State | 1.6 (0.7- 4.1) | 0.40 (0.14- 1.18) | 0.096 | 0.16 (0.04- 0.69) | 0.014 |
| KwaZulu Natal | 4.6 (3.0- 7.1) | 1.15 (0.56- 2.36) | 0.695 | 0.91 (0.42- 1.99) | 0.815 |
| Limpopo | 4.2 (1.4- 11.8) | 1.04 (0.30- 3.63) | 0.947 | 1.38 (0.35- 5.08) | 0.625 |
| Mpumalanga | 3.6 (1.2- 10.8) | 0.90 (0.25- 3.26) | 0.871 | 0.65 (0.18- 2.31) | 0.506 |
| Northern Cape | 4.1 (1.4- 11.5) | 1.02 (0.29- 3.53) | 0.976 | 0.81 (0.22- 2.89) | 0.739 |
| North West | 2.9 (0.4- 15.2) | 0.84 (0.48- 1.44) | 0.517 | 1.11 (0.58- 2.15) | 0.749 |
| Western Cape | 3.9 (2.2- 6.7) | 0.98 (0.44- 2.14) | 0.925 | 0.59 (0.23- 1.50) | 0.270 |

**Supplementary Table S2: Factors associated with being syphilis positive among pregnant women attending a follow up visit in the 2019 ANC survey and had documented syphilis screening results in a model including the interaction term, South Africa, N= 19 795**

| **Variable** | **Multivariable logistic regression**  **aOR (95% CI)** | **p-value** |
| --- | --- | --- |
| *Age category (years), n (%)* |  |  |
| 15-19 | 1.00 |  |
| 20- 24 | 1.85 (1.34- 2.55) | <0.001 |
| 25- 29 | 1.51 (1.06- 2.13) | 0.021 |
| 30- 34 | 1.35 (0.93- 1.96) | 0.115 |
| 35- 49 | 1.16 (0.77- 1.76) | 0.471 |
| *Population group* |  |  |
| Black | 1.00 |  |
| Mixed race | 1.33 (1.02- 1.75) | 0.036 |
| Other^#^ | 0.40 (0.12- 1.36) | 0.142 |
| *Education* |  |  |
| None/primary | 1.55 (1.11- 2.17) | 0.014 |
| Secondary | 1.28 (0.99- 1.67) | 0.062 |
| Tertiary | 1.00 |  |
| *Relationship with partner who fathered current pregnancy* |  |  |
| Married | 1.00 |  |
| Living together as married | 1.25 (0.96- 1.62) | 0.095 |
| Not living together but in a relationship | 1.41 (1.10- 1.81) | 0.007 |
| No relationship | 1.81 (1.23- 2.66) | 0.003 |
| *Geo-location* |  |  |
| Rural | 1.00 |  |
| Peri-urban | 1.19 (0.93- 1.51) | 0.163 |
| Urban | 1.20 (0.88- 1.63) | 0.242 |
| *Parity* |  |  |
| 0 | 1.00 |  |
| 1 | 0.83 (0.61- 1.13) | 0.227 |
| ≥2 | 0.80 (0.57- 1.13) | 0.209 |
| *Gravidity* |  |  |
| <2 | 1.00 |  |
| ≥2 | 1.61 (1.16- 2.24) | 0.004 |
| *HIV/ART status by province* |  |  |
| *Eastern Cape* |  |  |
| HIV negative | 1.00 |  |
| HIV positive , on ART | 1.74 (1.22- 2.49) | 0.002 |
| HIV positive , not on ART | 5.38 (3.01- 9.62) | <0.001 |
| *Free State* |  |  |
| HIV negative | 1.00 |  |
| HIV positive , on ART | 2.16 (1.45- 3.21) | <0.001 |
| HIV positive , not on ART | 3.65 (1.56- 8.52) | 0.003 |
| *Gauteng* |  |  |
| HIV negative | 1.00 |  |
| HIV positive , on ART | 3.76 (2.32- 6.07) | <0.001 |
| HIV positive , not on ART | 3.65 (1.56- 8.52) | 0.003 |
| *KwaZulu Natal* |  |  |
| HIV negative | 1.00 |  |
| HIV positive , on ART | 2.47 (1.90- 3.21) | <0.001 |
| HIV positive , not on ART | 0.84 (0.25- 2.85) | 0.774 |
| *Limpopo* |  |  |
| HIV negative | 1.00 |  |
| HIV positive , on ART | 9.01 (4.06- 20.00) | <0.001 |
| HIV positive , not on ART | 5.20 (0.93- 29.01) | 0.060 |
| *Mpumalanga* |  |  |
| HIV negative | 1.00 |  |
| HIV positive , on ART | 3.03 (1.60- 6.23) | 0.001 |
| HIV positive , not on ART | 1.36 (0.31- 5.96) | 0.681 |
| *Northern Cape* |  |  |
| HIV negative | 1.00 |  |
| HIV positive , on ART | 2.40 (1.26- 4.56) | 0.002 |
| HIV positive , not on ART | 1.91 (0.37- 9.90) | 0.440 |
| *North West* |  |  |
| HIV negative | 1.00 |  |
| HIV positive , on ART | 3.12 (1.49- 6.54) | 0.003 |
| HIV positive , not on ART | 3.65 (1.56- 8.52) | 0.003 |
| *Western Cape* |  |  |
| HIV negative | 1.00 |  |
| HIV positive , on ART | 1.70 (0.99- 2.91) | 0.052 |
| HIV positive , not on ART | 4.09 (1.78- 9.41) | 0.001 |
| *HIV negative* |  |  |
| Gauteng | 1.00 |  |
| Eastern Cape | 1.95 (1.21- 3.14) | 0.006 |
| Free State | 2.49 (1.56- 3.98) | <0.001 |
| KwaZulu-Natal | 1.92 (1.21- 3.05) | 0.006 |
| Limpopo | 0.56 (0.26- 1.19) | 0.129 |
| Mpumalanga | 1.09 (0.61- 1.96) | 0.796 |
| Northern Cape | 2.08 (1.21- 3.55) | 0.008 |
| North West | 0.89 (0.40- 1.99) | 0.784 |
| Western Cape | 0.61 (0.99- 2.63) | 0.057 |
| *HIV positive on ART* |  |  |
| Gauteng | 1.00 |  |
| Eastern Cape | 0.91 (0.61- 1.34) | 0.623 |
| Free State | 1.43 (0.97- 2.10) | 0.069 |
| KwaZulu Natal | 1.26 (0.92- 1.74) | 0.153 |
| Limpopo | 1.34 (0.78- 2.30) | 0.295 |
| Mpumalanga | 0.88 (0.42- 1.86) | 0.738 |
| Northern Cape | 1.32 (0.75- 2.33) | 0.329 |
| North West | 0.74 (0.43- 1.27) | 0.275 |
| Western Cape | 0.73 (0.44- 1.21) | 0.220 |
| *HIV positive not on ART* |  |  |
| Gauteng | 1.00 |  |
| Eastern Cape | 2.89 (1.12- 7.45) | 0.029 |
| Free State | 2.49 (1.56- 3.98) | <0.001 |
| KwaZulu Natal | 0.44 (0.11- 1.84) | 0.261 |
| Limpopo | 0.79 (0.14- 4.66) | 0.799 |
| Mpumalanga | 0.41 (0.07- 2.25) | 0.304 |
| Northern Cape | 1.09 (0.15- 7.69) | 0.932 |
| North West | 0.89 (0.40- 1.99) | 0.784 |
| Western Cape | 1.81 (0.59- 5.58) | 0.303 |

**Supplementary Table S3:** **The fourth generation serology platforms used for HIV testing in the 2019 antenatal survey**

| **Province** | **HIV screening test** | **HIV confirmatory test** |
| --- | --- | --- |
| Eastern Cape | Abbott Architect HIV Ag/Ab I1000sr | COBAS 6000 c module |
| Limpopo/North West | Cobas HIV Combi PT ( E 601 Roche) | Architect HIV Ag/Ab combo (Architect Abbott) |
| Western and Northern Cape | Roche Cobas 6000 e601 HIV Combi PT Elecsys (Ag/Ab) | Abbott Architect HIV Ag/Ab Combo kit |
| Mpumalanga | HIV Combi PT 4th generation - Roche Cobas 6000 | CHIV Siemens Centaur 4th generation |
| Free State | Roche Cobas 6000 e 601 | Minividas HIV quick DUO |
| KwaZulu-Natal | Abbott Architect HIV Antigen/Antibody Combo | Roche HIV Combi PT |
| Gauteng | Abbott Architect I1000SR HIV Ag/Ab combo | Diasorin Murex Ag/Ab Combination EIA |
